# Resistance priming strategies in professional Spanish soccer: A survey study into the practices and perceptions of strength and conditioning coaches

**DOI:** 10.5114/biolsport.2026.156228

**Published:** 2025-12-04

**Authors:** Enrique Cañadas-García, Alfonso de la Rubia, Anthony Weldon, Jaime González-García, Verónica Giráldez-Costas, Carlos García-Sánchez

**Affiliations:** 1 Deporte y Entrenamiento Research Group, Departamento de Deportes, Facultad de Ciencias de la Actividad Física y del Deporte (INEF), Universidad Politécnica de Madrid, C/Martín Fierro 7, 28040 Madrid, Spain; 2 Research for Athlete and Youth Sport Development (RAYSD) Lab, Faculty of Health, Education and Life Sciences, Birmingham City University, Birmingham B15 3TN, UK; 3 Aston Villa Foundation, Aston Villa Football Club, Birmingham, B6 6HE, UK; 4 Exercise and Sport Sciences, Faculty of Health Science, Universidad Francisco de Vitoria, 28223 Pozuelo, Spain; 5 Strength Training and Neuromuscular (STreNgthP) Research Group. Camilo José Cela University. Faculty of Health Science Madrid, Spain

**Keywords:** Strength, Soccer, Priming, Performance, Coaching practices

## Abstract

This cross-sectional survey study investigated the practices and perceptions of strength and conditioning (S&C) coaches working in professional Spanish soccer concerning resistance priming strategies. Twenty-four S&C coaches (age: 30.6 ± 5.3 years; professional experience: 7.7 ± 3.5 years) participated in this study. The survey comprised four sections: 1) coaches’ information; 2) perceptions about resistance priming; 3) programming variables; and 4) opinions. Results showed that S&C coaches predominantly used resistance priming strategies 24 h (79%) or 24–48 h (21%) before a match. The most frequently used exercises during resistance priming sessions were isometric exercises (25%), followed by traditional strength exercises heavy load (85% 1RM) (22%), traditional strength moderate load (60–85% 1RM) (17%), ballistic exercises (17%), traditional strength light load (< 60 % 1RM) (11%), sprint (4%), and weightlifting (4%). This paper analyses survey responses from S&C coaches in soccer, comparing their reported practices and perceptions with the findings of existing empirical research. This allows us to examine whether their methods are consistent with research-based approaches, or if they deviate towards alternative methods. This information can help professionals design more effective training programmes.

## INTRODUCTION

Strength is a key determinant of athletic performance [1]. Stronger athletes tend to show greater force outputs and movement efficiency, which may reduce associated fatigue [2] and injury risk [3, 4, 5, 6]. A recent systematic review concluded that strength training in soccer players, especially using heavy loads of at least 85% of the onerepetition maximum (1RM), significantly improves sprinting and jumping abilities [7]. However, recent research has also shown that ballistic exercises involving light to moderate loads can be effective [8]. Considering the benefits of strength training in soccer players, it is important to consider how such training can be incorporated throughout the competitive season. Most likely, these programmes in team sport focus on increasing intensity rather than total volume. The use of microdosing strategies could be considered as a means to induce a post-activation potentiation (PAPE) or resistance priming (RP) effects [9]. The implementation of strength training programmes can lead to significant improvements in both maximal strength and short sprint performance in soccer [10]. Pre-competition activation protocols are commonly used to enhance athletic performance, serving as the final exercise-based intervention to increase muscle force before a match day [11]. These may include PAPE, which is generally performed just before competition and RP, which can take place 1–48 hours before competition [12, 13]. The use of RP sessions aims to provide delayed potentiation (enhancement) of strength, power and other neuromuscular parameters without extending recovery time [12, 13, 14, 15], to improve key athletic abilities (e.g., jumping, sprinting, and changing of direction). In elite soccer competition, RP sessions have also been shown to influence players’ physical performance, improving total distance covered and high-intensity accelerations and decelerations [16]. Moreover, this strategy can positively influence athletes’ perceived readiness and confidence before competition [17]. Likewise, RP may be applied to maintain adequate levels of strength and power during extensive competitive periods in team sports, in which S&C coaches, sports coaches, and athletes typically struggle to provide efficient and effective programming due to such limitations [10, 18].

Accordingly, S&C coaches administering RP sessions should know the influence of volume, load, exercise task, intensity, movement intent, and recovery period [19]. While many studies describe RP as involving low-volume strength stimuli performed at moderate to heavy loads and maximum intended velocity (e.g., 1–3 sets, 1–3 repetitions, ≥ 85% 1RM) [10, 12, 20], other protocols involve heavy resisted sprints using sled load equivalent to 100% of the athlete’s body mass [14], ballistic exercises with moderate loads (40% 1RM) [9, 20], traditional strength exercises (e.g., deadlift, squat, bench press, etc) [14, 21, 22, 23], weightlifting movements [24, 25], or ballistic exercises [26]. From a physiological perspective, various mechanisms underlying the improvement in athletic performance following RP sessions exist, although these are not yet fully understood. The proposed mechanism behind these effects is increased neural activation, which subsequently improves central nervous system function and muscle fibre recruitment [19]. Other potential mechanisms include greater neuromuscular drive at a lower energy cost through maximal isometric exercise [27]. In addition, elevated testosterone levels may contribute to this process, as they may be associated with enhanced readiness [28]. However, this remains a theoretical explanation.

Recently, descriptive studies have been carried out to analyse the general practices and perceptions of soccer S&C coaches [18, 29, 30]. Despite the increasing interest in optimising pre-competition strategies, limited research has investigated the practices of S&C coaches specifically concerning RP [31, 32]. A survey reported that 59% of the professionals, working in roles involving resistance training for high-performance athletes, introduced RP 0–8 hours before the competition. Professionals were allowed to select multiple options regarding both the type of exercise and intensity used. The most commonly selected lower-body exercises included unloaded lunges (87%), loaded lunges (60%), partial (41%) and full squat (18%), olympic lift variation (11%), and other lower body exercises (11%). Reported intensities varied considerably, with professionals using: ≥ 85% 1RM (22%), 84–70% 1RM (38%), 69–50% 1RM (38%), 49–30% 1RM (24%) and < 30% 1RM (29%) [32]. Similarly, another study indicated that the majority of practitioners, currently employed by professional sporting clubs and responsible for prescribing priming sessions to athletes (e.g., athletics, cycling, rugby sevens, weightlifting, and soccer), surveyed on workload selection opted for light loads (≤ 30% 1RM) and reported variability in exercise selection (e.g., resistance exercise, sprint, ballistic exercises) [32]. Therefore, given the limited consistency in terms of RP across different studies and the absence of literature, this study investigated the practices and perceptions of soccer S&C coaches concerning their use of RP within their roles in professional Spanish soccer.

## MATERIALS AND METHODS

### Participants

Twenty-four male Spanish S&C soccer coaches (age: 30.62 ± 5.32 years; professional experience: 7.7 ± 3.5 years) participated in this study. Participants actively coached professional soccer players within the Spanish third and fourth divisions (e.g., First RFEF and Second RFEF), equating to tier three in the established participant classification framework, who compete at the national level [33].

### Procedures

The methods and anonymous online survey used in this study were adapted from previous research [17, 32, 34]. Furthermore, the survey was assessed against the Checklist for Reporting Results of Internet E-Surveys (CHERRIES) [35] and the Checklist for Reporting of Survey Studies (CROSS) [36] (see appendices 2 and 3). The survey was developed using the survey application Google Forms (Alphabet Inc., Mountain View, CA). The content validity of the survey was assessed by a panel of 16 experts, 14 of whom held a PhD in sports science and 2 elite soccer coaches. Experts evaluated each item for relevance and wording using a Likert scale from 1 to 10, where one represents the minimum presence of the attribute and ten represents the maximum. In addition, each question was analysed qualitatively so that expert judges could add suggestions or comments. Minor wording adjustments were made based on their feedback. Question Q13, for example, was revised from “select a reason” to “select the reason for your assessment of the RP effectiveness in producing a performance effect”. The final survey consisted of 38 fixed responses and one open-ended questions across four sections: a) coaches information (12 questions), b) perceptions about resistance priming (6 questions), c) programming variables (time interval between RP and the match, exercise type and intensity, volume and load control) (20 questions) and d) opinions (1 question) (see Appendix 1).

The survey was distributed via the corresponding author’s social networks (e.g., LinkedIn, WhatsApp, Instagram) and remained open from 1^st^ November 2024 to 30^th^ November 2024. Before completing the survey, all participants were informed of the procedures and aims of the study. They gave their informed consent, and the confidentiality of their information was ensured during their participation. For S&C coaches, the inclusion criteria were: (a) currently involved in competitive professional soccer, coaching players who have achieved at least tier 3 [33], and (b) taking direct responsibility for planning gym-based S&C programmes, including designing and supervising strength sessions for players. All participants received an explanation of the study’s purpose and signed an informed consent form. The participation was strictly confidential and voluntary. The project and the scientific use of the data were approved by the Ethics Committee of the Polytechnic University of Madrid (FDRED00000-DML-DATOS-20230609) in compliance with the Declaration of Helsinki.

### Statistical analysis

The psychometric properties of the survey were assessed for content validity using Aiken’s V coefficient [36]. The Visual Basic program developed by Merino & Livia [37] was used to perform the analysis, considering values ≥ 0.70 as an adequate value with two confidence intervals of 95% [36, 37, 38, 39]. Responses to the survey were downloaded into a customized Microsoft Excel Spreadsheet (Microsoft, Redmond, WA, version 16.68, USA). Descriptive statistics are presented as mean values and standard deviations (X ± SD). Fixed response questions were assessed using relative and absolute frequency analysis. Data analysis was performed using SPSS for Windows (Version 26, IBM Corp., Armonk, NY, USA).

## RESULTS

Table 1 illustrates the Aiken V coefficient values for each questions and the confidence intervals, according to their relevance, definition and importance. All questions have obtained validity values above 0.70, thus being considered adequate.

**TABLE 1:** Relevance and Wording Aiken’s V values of survey sections.

Sections		Relevance			Wording		

		Aiken’s V	Mean ± SD	IC (95%)	Aiken’s V	Mean ± SD	IC (95%)
**Coaches information**	Q1	0.88	8.88 ± 1.54	0.81–0.95	0.95	9.56 ± 0.81	0.90–0.98
	Q2	0.88	8.88 ± 1.78	0.81–0.92	0.96	9.63 ± 0.72	0.91–0.98
	Q3	0.89	9.00 ± 1.71	0.83–0.93	0.83	8.44 ± 2.16	0.76–0.88
	Q4	0.92	9.31 ± 1.30	0.87–0.96	0.96	9.63 ± 0.72	0.91–0.98
	Q5	0.92	9.25 ± 1.57	0.86–0.95	0.90	9.13 ± 1.36	0.84–0.94
	Q6	0.92	9.25 ± 1.18	0.86–0.95	0.84	8.56 ± 1.75	0.77–0.89
	Q7	0.93	9.38 ± 1.15	0.88–0.96	0.81	8.31 ± 1.99	0.74–0.87
	Q8	0.94	9.44 ± 1.21	0.89–0.97	0.91	9.19 ± 1.11	0.85–0.95
	Q9	0.94	9.44 ± 1.21	0.89–0.97	0.93	9.38 ± 1.15	0.88–0.96
	Q10	0.88	8.94 ± 2.14	0.82–0.92	0.87	8.81 ± 2.20	0.80–0.91
	Q11	0.97	9.69 ± 0.60	0.92–0.99	0.87	8.81 ± 2.01	0.80–0.91

**Perceptions about resistance priming**	Q12	0.92	9.25 ± 1.34	0.86–0.95	0.81	8.31 ± 2.57	0.74–0.87
	Q13	0.90	9.13 ± 1.50	0.84–0.94	0.86	8.75 ± 1.75	0.80–0.91
	Q14	0.94	9.44 ± 0.89	0.89–0.97	0.72	7.50 ± 2.88	0.64–0.79
	Q15	0.85	8.69 ± 2.47	0.79–0.90	0.85	8.63 ± 1.78	0.78–0.90
	Q16	0.83	8.44 ± 2.42	0.76–0.88	0.83	8.50 ± 2.42	0.76–0.89
	Q17	0.70	7.31 ± 3.14	0.62–0.77	0.78	8.00 ± 2.68	0.70–0.84

**Programming variables**	Q18	0.90	9.06 ± 1.69	0.84–0.94	0.82	8.38 ± 2.03	0.75–0.87
	Q19	0.90	9.06 ± 2.24	0.84–0.94	0.85	8.63 ± 2.70	0.78–0.90
	Q20	0.91	9.19 ± 1.42	0.85–0.95	0.85	8.69 ± 1.58	0.79–0.90
	Q21	0.98	9.81 ± 0.40	0.94–0.99	0.93	9.38 ± 0.96	0.88–0.96
	Q22	0.95	9.56 ± 0.89	0.90–0.98	0.82	8.38 ± 2.33	0.75–0.87
	Q23	0.93	9.38 ± 0.96	0.88–0.96	0.85	8.63 ± 1.63	0.78–0.90
	Q24	0.88	8.88 ± 1.71	0.81–0.92	0.79	8.13 ± 2.09	0.72–0.85
	Q25	0.92	9.25 ± 1.39	0.86–0.95	0.87	8.81 ± 1.60	0.80–0.91
	Q26	0.88	8.88 ± 2.09	0.81–0.92	0.83	8.44 ± 1.97	0.76–0.88
	Q27	0.94	9.44 ± 1.09	0.89–0.97	0.92	9.31 ± 1.35	0.87–0.96
	Q28	0.90	9.13 ± 1.45	0.84–0.94	0.89	9.00 ± 1.59	0.83–0.93
	Q29	0.94	9.44 ± 0.96	0.89–0.97	0.90	9.06 ± 1.39	0.84–0.94
	Q30	0.90	9.13 ± 1.45	0.84–0.94	0.88	8.94 ± 1.73	0.82–0.92
	Q31	0.89	9.00 ± 1.63	0.83–0.93	0.88	8.94 ± 1.73	0.82–0.92
	Q32	0.93	9.38 ± 1.15	0.88–0.96	0.90	9.13 ± 1.31	0.84–0.94
	Q33	0.94	9.50 ± 0.89	0.89–0.97	0.91	9.19 ± 1.05	0.85–0.95
	Q34	0.94	9.50 ± 0.89	0.89–0.97	0.90	9.13 ± 1.15	0.84–0.94
	Q35	0.88	8.94 ± 2.32	0.82–0.92	0.85	8.69 ± 2.41	0.79–0.90
	Q36	0.88	8.88 ± 2.39	0.81–0.92	0.83	8.50 ± 2.37	0.76–0.89
	Q37	0.94	9.50 ± 0.89	0.89–0.97	0.94	9.44 ± 1.09	0.89–0.97
	Q38	0.97	9.75 ± 0.58	0.93–0.99	0.88	8.88 ± 2.31	0.81–0.92

**Opinions**	Q39	0.89	9.00 ± 2.25	0.83–0.93	0.92	9.25 ± 1.24	0.86–0.95

### Coaches information

All descriptive results are reported as absolute (n) and relative values (%). Participants in this study worked across the Spanish third 11 (46%) and fourth divisions 13 (54%). Regarding academic qualifications, most held a master’s degree in sports science or related field 19 (79%), a PhD 4 (17%) and a bachelor’s degree in sport science 1 (4%). The majority of S&C coaches held a professional football qualification: UEFA B 13 (54%), UEFA A 7 (29%), and UEFA C 3 (13%), while a small proportion had no UEFA qualification 1 (4%). Few 4 (18%) participants held recognised S&C qualifications, which were obtained from the National Strength and Conditioning Association (NSCA) 3 (13%) and the United Kingdom Strength and Conditioning Association (UKSCA) 1 (4%).

### Perceptions about resistance priming

Participants reported that RP was effective 10 (42%), very effective 6 (25%), moderately effective 5 (21%) and slightly effective 3 (12%) in having a positive effect on performance in competition. Table 2 shows the frequency of responses regarding the effectiveness of RP exercise on neuromuscular performance, mental preparation and perception of recovery. Regarding the reasons for introducing this strategy into the training plan, a significant number of coaches 13 (54%) believe in the effectiveness of the strategy, while 11 (46%) report that their players have reported improvements after introducing the strategy.

**TABLE 2:** Absolute and relative frequency (n and %) of responses regarding to the effectiveness of resistance priming exercises (*n* = 24).

	Not effective n (%)	Slightly effective n (%)	Moderately effective n (%)	Effective n (%)	Very effective n (%)
Neuromuscular performance	0 (0)	0 (0)	3 (13)	13 (54)	8 (33)
Mental preparation	0 (0)	2 (8)	6 (25)	11 (46)	5 (21)
Perception of recovery	3 (12)	4 (17)	6 (25)	4 (17)	7 (29)

**TABLE 3:** Absolute and relative frequency (n and %) of responses regarding the reason for the timing of the strategy implementation (*n* = 24).

	Absolute (n)	Relative (%)
I don’t have the opportunity to introduce it on the same day of the match	9	38
I follow the trend of the latest scientific publications	8	33
The players report a preference for implementing it 24 hours before the competition	3	13
Moving it away from the competition by > 24 hours favour complete recovery	2	8
The players report a preference for doing it on the same day as the competition	2	8

Concerning the biggest obstacles faced when implementing RP participants responded players’ lack of experience 9 (38%), lack of time, space or material and/or playing away from home 8 (33%) and no difficulties 7 (29%).

### Programming variables

#### Time interval between the RP and the match

All S&C coaches 24 (100%) implemented RP within 0–48 hours before competition, with most implementing it 24 hours before 19 (79%) and a smaller proportion between 24–48 hours prior 5 (21%).

### Exercise type and intensity

**FIG. 1:**
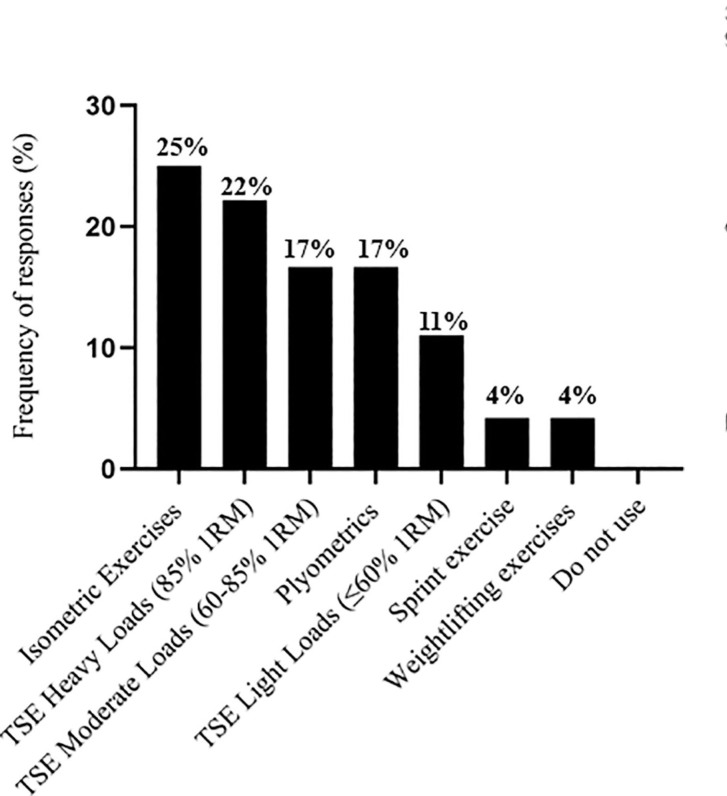
Selected training stimulus during priming sessions by soccer strength and conditioning coaches. Traditional strength exercises (TSE).

**FIG. 2:**
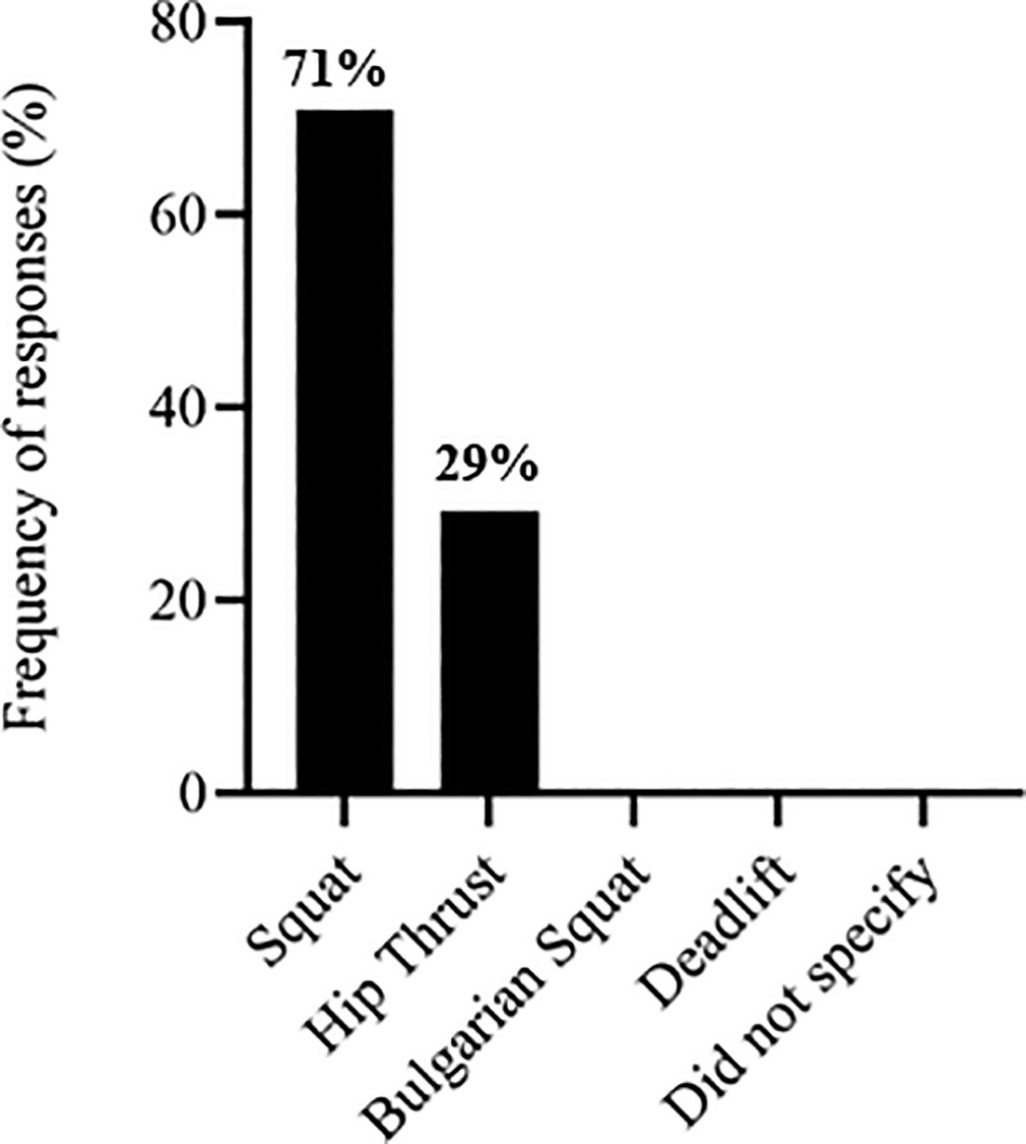
Traditional strength exercises used by soccer strength and conditioning coaches.

**FIG. 3:**
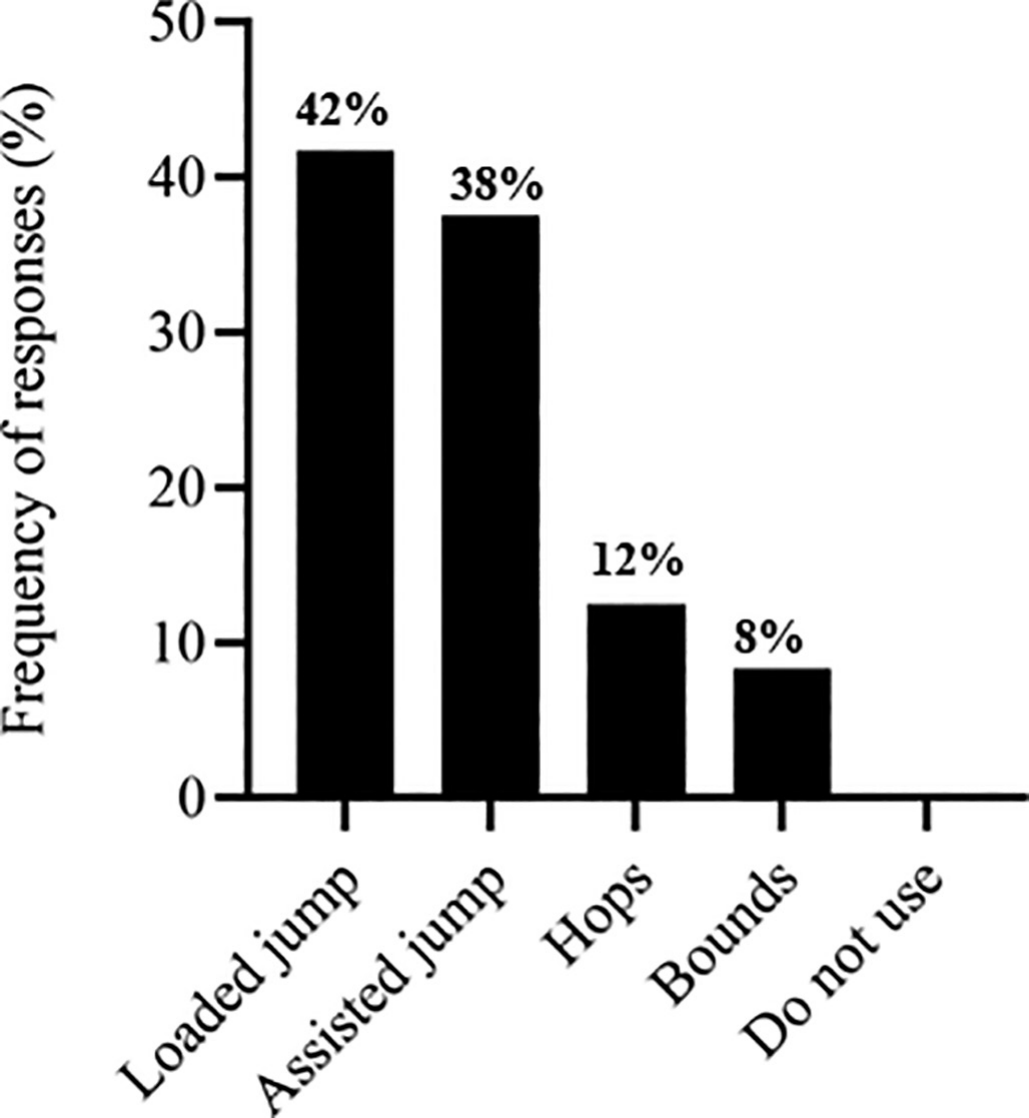
Ballistic exercises used by soccer strength and conditioning coaches.

**FIG. 4:**
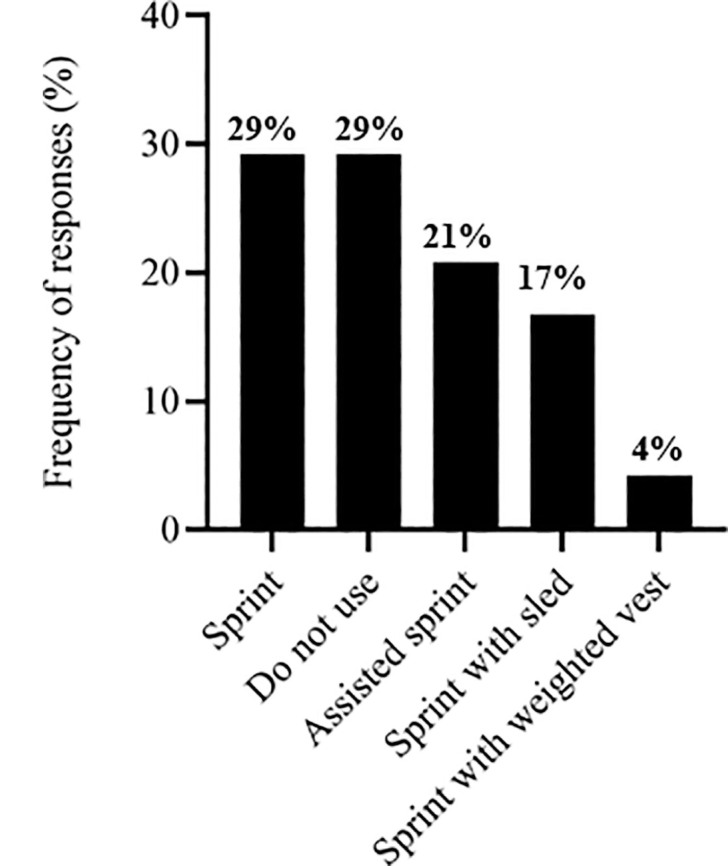
Sprint exercises used by soccer strength and conditioning coaches.

**FIG. 5:**
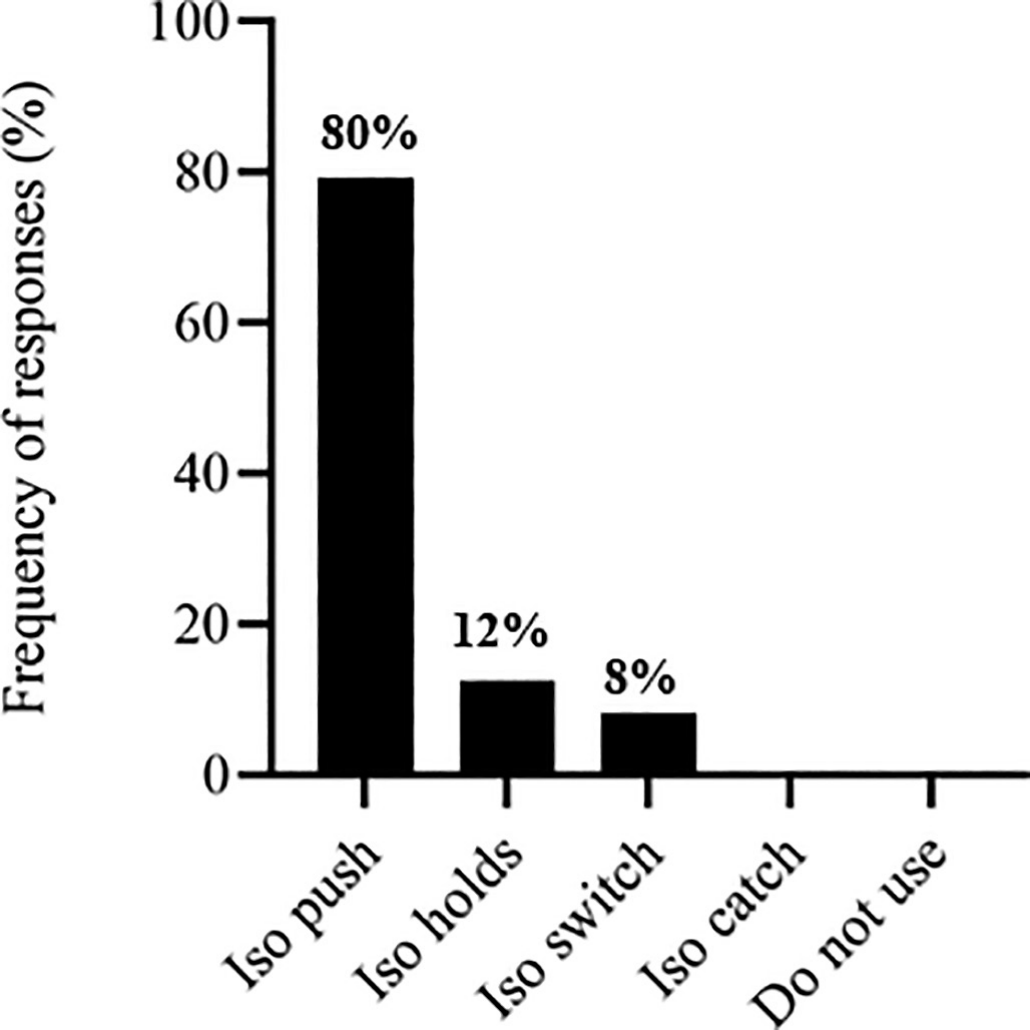
Isometric exercises used by soccer strength and conditioning coaches.

The S&C coaches’ responses regarding exercise selection across different training modalities. Figure 1 depicts responses related to the training stimulus. Figure 2 presents the selection of traditional strength exercises, followed by ballistic exercises (Figure 3), sprint exercises (Figure 4), and isometric (ISO) strength training (Figure 5).

Table 4 presents the ranking of the five most important exercises used by S&C coaches in their RP sessions. Each coach was asked to rate the perceived effectiveness of the PR sessions on a scale from 1 to 5, with 1 being the most important and 5 being the least. Rankings were calculated by determining the absolute and relative frequencies of each exercise in each ranking position. In cases where multiple exercises received the same ranking frequency, no additional weighting was applied, and ties were treated as equal contributions to the overall frequency counts. This approach provided a clear visualisation of the exercises that were most consistently prioritised by coaches.

**TABLE 4:** Absolute and relative frequency (n and %) of ranking of the exercises used in the priming sessions (*n* = 24).

	Order of importance				
**Exercises**	**1 n (%)**	**2 n (%)**	**3 n (%)**	**4 n (%)**	**5 n (%)**
Ballistic exercises	11 (46)	10 (42)	3 (12)	0 (0)	0 (0)
Isometric exercises	5 (21)	8 (33)	5 (21)	5 (21)	1 (4)
Traditional strength exercises	4 (17)	8 (33)	6 (25)	3 (13)	3 (12)
Sprint	1 (4)	4 (17)	8 (33)	8 (33)	3 (13)
Weightlifting exercises	1 (4)	5 (21)	4 (16)	10 (42)	4 (17)

### Volume

Table 5 shows the frequency of responses regarding the number of sets, repetitions and minutes for each exercise used in the priming sessions by S&C coaches.

**TABLE 5:** Absolute and relative frequency (n and %) of responses regarding the number of sets, repetitions and minutes for each exercise used in the priming sessions (*n* = 24).

Number of sets for each exercises		**1 (%)**	**2 (%)**	**3 (%)**	**4 (%)**	**+5 (%)**
	Traditional strength	1 (4)	14 (58)	9 (38)	0 (0)	0 (0)
	Ballistic exercises	1 (4)	16 (67)	5 (21)	1 (4.)	1 (4)
	Isometric	2 (8)	12 (50)	6 (25)	3 (13)	1 (4)
	Sprint	6 (25)	8 (33)	3 (13)	4 (17)	3 (12)

Number of repetitions for each exercises		**1 (%)**	**2 (%)**	**3 (%)**	**4 (%)**	**+5 (%)**
	Traditional strength	1 (4)	0 (0)	6 (25)	12 (50)	5 (21)
	Ballistic exercises	0 (0)	1 (4)	6 (25)	3 (13)	14 (58)

Time to rest for each exercises (minutes)		**1 (%)**	**2 (%)**	**3 (%)**	**4 (%)**	**Other**
	Traditional strength	2 (8)	14 (58)	7 (29)	1 (4)	0 (0)
	Ballistic exercises	6 (25)	13 (54)	4 (17)	1 (4)	0 (0)
	Isometric	8 (33)	10 (42)	4 (17)	2 (8)	0 (0)
	Sprint	5 (21)	8 (33)	7 (29)	4 (17)	0 (0)

### Load control

The majority of S&C coaches 21 (88%) monitored the intensity during the implementation of the RP. When asked about the methods used, they reported that using velocity-based training (VBT) 13 (54%), verbal communication 6 (25%), the perceived exertion scale 3 (13%) and neuromuscular performance tests 2 (8%).

## DISCUSSION

This study surveyed the practices and perceptions of S&C coaches working in professional Spanish soccer concerning RP. The main findings indicate that: (1) All participants implemented RP, which was predominantly delivered 24 hours (79%) or 24–48 hours (21%) before the competition; (2) S&C coaches perceived RP to have a positive effect on performance in competition, with the majority describing it as effective (42%), followed by very effective (25%), moderately effective (21%) and slightly effective (12%); (3) The training stimulus that S&C coaches most commonly used for RP was isometric (25%), followed by traditional strength exercises heavy load (85% 1RM) (22%). The results confirm that S&C coaches consider RP a relevant strategy, recognising its effectiveness in improving neuromuscular performance (54%). Similarly, previous research has shown that this strategy is effective for specific neuromuscular parameters, such as increased jumping and sprinting, as well as improvements in movement velocity and power production [14, 21, 40, 41]. Holmberg et al. [32] suggest that the most consistent evidence for RP comes from studies of highly trained athletes, where low-volume, high-intensity resistance exercise performed with maximal intent and within six hours of competition has shown the greatest performance benefits. An additional comment from S&C coaches indicated that *“Players with higher strength values and more experience in strength training will benefit much more from PR sessions”.* In this regard, 54% of coaches reported using RP because they believe it is effective, while 46% noted that their players had reported perceived improvements after implementing the strategy. Likewise, previous studies showed that RP was considered beneficial for speed and power, strength, and agility [32], based on a sample of 69 experienced S&C professional coaches. Despite its potential to potentiate performance, it has been observed that RP sessions can produce individual differences [18]. This suggests that S&C coaches’ perceptions may be based on the individual responses of their players.

On the other hand, the S&C coaches considered its benefits for mental preparation (21%); these findings were similar to those found by González-García et al. [14], where they observed a higher readiness in the priming condition at six hours compared to the pre-priming condition, using psychological readiness as one of the indicators measured. As for the perception of recovery, responses were more heterogeneous (Table 2), where only 29% of coaches considered PR to be very effective in producing a positive effect on this variable. Similarly, Pino-Mulero et al. [15] reported no improvement in perceived recovery 24 hours after priming in soccer players.

Strength and conditioning coaches had difficulties implementing RP due to the players’ lack of experience, time, space or equipment and/or playing away from home. However, some coaches had no difficulties at all. These data differ slightly from those obtained by Holmberg et al. [32], who conducted their study with a sample of 28 S&C professionals currently employed in Australia. In their study, one of the main challenges in implementing priming strategies was the difficulty of convincing coaches and athletes of their benefits. It is, therefore, important to educate coaches and players to better understand how the process of stimulus, response, and adaptation occurs concerning priming strategies, which may influence increased buy-in.

### Programming variables

#### Time interval between the RP and the match

Most S&C coaches (79%) introduced RP 24 hours before the match, while the rest (21%) introduced it 24–48 hours before the competition. The results of this survey are consistent with previous studies that have examined the effects of their interventions within the same 24 to 48-hour period [9, 20, 40]. Furthermore, our results reveal that none of the coaches implemented this strategy on match day despite previous research highlighting its potential benefits for increasing performance and showing an adequate recovery [16, 17, 19]. This lack of implementation appears to be mainly due to practical barriers, such as the lack of opportunity to apply the strategy on match day (38%) and the desire to follow the most recent scientific recommendations (33%) (Table 4). Additionally, it is important to explore innovative solutions that can bridge this gap and facilitate the implementation of RP on match day. For example, one practical approach could be to schedule team travel the day before the match and conduct RP sessions on the morning of the match.

#### Exercise type and intensity

One of the training stimuli used to implement RP is traditional strength exercise performed at different intensities (Figure 1). Research conducted in controlled laboratory settings supports this approach, demonstrating that traditional strength exercises performed at 80% 1RM elicit significant neuromuscular benefits [14, 23, 31]. However, it differs from the findings by Holmberg et al. [32], where 80% of the participants used light loads (≤ 30% 1RM). These discrepancies in the application of intensities during priming sessions may be attributed to the varying recovery time required based on load intensity [9] and players’ strength levels [41]. Nevertheless, Loturco et al. [42] obtained similar responses in physical performance parameters when comparing the effects of light-load and heavy-load jump squats as RP on Olympic women’s rugby sevens players, although heavy loads were associated with higher perceived exertion. Consequently, practitioners may tailor the training stimulus load according to the available recovery period before competition, contributing to inconsistencies in load intensity selection and its subsequent effects on performance.

On the other hand, the fact that a significant number of S&C coaches (46%) considered ballistic exercises to be very important compared to other types of exercises (Table 5) suggests that the use of ballistic exercises may be a more specific stimulus for soccer players due to their nature of producing force at high velocities [43]. These findings are similar to those found in the study of Nishioka & Okada [8], who compared ballistic exercise with heavy resistance and found that ballistic exercise was most effective in improving countermovement jump (CMJ) after 24 hours. Additionally, 33% of coaches regarded isometric exercises as a key training stimulus. However, despite the preference for these types of training stimuli, Harrison et al. [22] reported limited benefits when implementing lowload squat jumps or maximal isometric exercises as priming strategies. Overall, traditional strength exercises are the most commonly used by professionals, but it is difficult to determine the intensity. Holmberg et al. [32] suggest that exercise and intensity selection were based on the comfort of the coach and athlete and, familiarity with the movements. One possible solution would be to standardise the methods used to quantify external load. For example, mean propulsive velocity could be used instead of 1RM estimation to avoid associated risks. In line with this Dhahbi et al. [44] found that 4–6RM tests provide a reliable and practical indicator for 1RM testing in trained male athletes.

The most commonly used traditional strength exercise was the squat (71%) (Figure 2), similar to other studies that selected this exercise for their research [14, 21, 40]. Loaded jumps are extensively employed by S&C coaches (42%) (Figure 3). They also used, to a similar extent, assisted ballistic (38%), hops (12%) and bounds (8%). Likewise, previous research has observed that 60% of practitioners chose loaded jumps to prescribe resistance training programs for high-performance athletes [31]. Strength and conditioning coaches reported diverse opinions regarding the type of sprint exercise used (Figure 4), including a lack of knowledge about whether sprint exercise was beneficial as a priming strategy. However, different research has found positive effects in implementing RP sessions [21, 45].

### Volume

Various responses were found for different training parameters (sets, repetitions, rest time). Approximately 50% of S&C coaches selected 2 sets per exercise for traditional strength exercises, isometrics and ballistic. Similarly, previous research has observed that participants selected an average of 2.8 sets per exercise [31]. However, greater variability was observed in the selection of sprint volume, with coaches using 2 sets (33%), 1 set (25%), 4 sets (17%), +5 sets (17%) or 3 sets (13%). In contrast, studies incorporating this stimulus typically prescribe a total of 5–6 sets [26]. For the number of repetitions of traditional strength exercises, 50% of the S&C coaches selected 4 repetitions and 25% of them 3 repetitions. Our results are in line with previous studies, where researchers suggest a volume of 3–5 repetitions per set [23, 31].

Regarding the number of repetitions in ballistic exercises, the majority of coaches concentrated between 6 repetitions (25%), 5 repetitions (25%) and 3 repetitions (25%). In previous research that used ballistic exercise, 3 repetitions were selected [22] or 4 repetitions [8]. A significant number of S&C (58%) in traditional strength exercises, (54%) in ballistic and (42%) in isometric exercise chose 2 minutes as the optimal rest time, while in sprint exercise, a wider variety was found, approximately 46% of coaches chose between 3 and 4 minutes to ensure a complete recovery. These parameters must be clarified, as the chosen exercise and intensity can influence the stimulus of the session, whereas the total volume influences the organism’s response [19]. Therefore, it seems clear that S&C coaches employ a low to medium number of repetitions (3–6) and a medium-long rest time (2–3 minutes), regardless of the exercise used.

### Load control

Lastly, S&C coaches reported that VBT (54%) was the most selected for monitoring intensity. Our results align with previous research, which examined the effects of this strategy by monitoring the velocity loss in the series (20%) [17]. Although VBT may be the ideal option to control training doses according to the individual characteristics of the subjects, the practical reality is much more complex and sometimes implementing this method is unfeasible. In this regard, Dhahbi et al. [44] defend to need to standardise the quantification of external load in training in order to improve the comparability of research and its practical application.

This study is subject to certain limitations. First, the small sample size (n = 24) limits the generalisability of the findings. Second, the recruitment strategy involved voluntary participation via social media networks, introducing the risk of selection bias. In future studies, a more formal approach could have been adopted, such as contacting the clubs directly or inviting the S&C coaches individually. Third, the use of social media for recruitment prevented us from determining the response rate, and random sampling was not employed. Finally, the study focused exclusively on the third and fourth divisions of Spanish soccer. Therefore, caution should be exercised when applying the results to other contexts or countries.

## CONCLUSIONS

Resistance priming is commonly implemented by S&C coaches in professional Spanish soccer teams, and has been reported to have an adequate perception of recovery and neuromuscular performance after implementation. Isometric exercises have emerged as the most commonly used for this strategy, closely followed by traditional strength and ballistic exercises. The majority of S&C coaches implemented it 24 hours before the competition. The S&C coaches included 2–3 sets of 3–4 repetitions per exercise. Ballistic exercises received a high score in the S&C coaches’ assessment and were the third option selected. The most commonly used exercises were the squat, ISO push, loaded jumps, and sprint without weights.
